# Perspectives on formation of medical cannabis market in Ukraine based on holistic approach

**DOI:** 10.1186/s42238-020-00044-y

**Published:** 2020-10-02

**Authors:** Nataliia Aliekperova, Кostyantyn Kosyachenko, Oleksandr Kaniura

**Affiliations:** 1 Department of Organization and Economics in Pharmacy, Bogomolets National Medical University, 13, T. Shevchenko blvd., Kyiv, 01601 Ukraine; 2 Bogomolets National Medical University, 13, T. Shevchenko blvd., Kyiv, 01601 Ukraine

**Keywords:** Medical cannabis, Market regulations, Pharmacy students, USSR, Former USSR countries, Ukraine

## Abstract

**Background:**

Nowadays, medical cannabis still remains inaccesible for patients in the former Union of Soviet Socialist Republics (USSR). Even registered medicinal products based on herbal or synthetic cannabinoids, like Sativex, are practically unavailable due to their high cost and narrow scope of application (for example, in Lithuania). However, before the absolute prohibition of medical cannabis in the USSR, in line with Single Convention of 1961, the State Pharmacopoeia of the USSR (eighth edition) published monographs on such medicinal products as “Herba *Cannabis indicae*”, “Extractum *Cannabis indicae* spissum” and “Fructus Cannabis”, which could be prescribed by physicians with precaution.

**Objectives:**

Formation of a holistic approach aimed at the creation of appropriate conditions for the development of medical cannabis market and the improvement of life quality and health of Ukrainian patients.

**Methods:**

We analyzed legislation and regulation mechanisms for medical cannabis in the USSR, and the present availability of these products for patients in the former USSR, such as Lithuania, Georgia, Estonia, Russian Federation, and Ukraine. Four hundred thirty-five Ukrainian pharmacy students participated in the quantitative analysis (a survey) that took place on April–May, 2019 at Bogomolets National Medical University (Kyiv, Ukraine). They were surveyed about legalization of medical cannabis in Ukraine, advisability of including cannabis and cannabinoids related data into educational programs, and other issues. Qualitative analysis we applied consisted of the stakeholder analysis and Strength, Weakness, Opportunity, and Threat (SWOT) analysis. We classified the key stakeholders into the patients, healthcare professionals, legislative and regulatory bodies, pharmaceutical companies, pharmacies, etc., and ranked them based on their power and interest in the development of potential medical cannabis market in Ukraine. We also identified their expectations and goals. SWOT analysis allows us to evaluate predictable risks and opportunities, as well as strong and weak aspects of the effective development of medical cannabis industry in Ukraine.

**Results:**

According to the survey among pharmacy students, about 80% support the legalization of cannabis for medical purposes. However, two-thirds of them think that there is a risk of illicit turnover. Nearly half of the respondents are not informed or poorly informed about cannabis therapeutic properties. At the same time, nearly 90% consider that materials on medicinal properties of cannabis and cannabinoids should be included in the training program. Nowadays, such stakeholders as legislative and regulatory bodies have the highest power over the development of potential medical cannabis market in Ukraine: more than two million Ukrainian patients still cannot access an effective cannabinoids based treatment. There are over 20 thousand children among them suffering from drug-resistant epilepsy due to the lack of adequate legislation. Moreover, a lot of stakeholders with high level of interest, such as growers, manufacturers of cannabidiol (CBD) products, pharmaceutical companies, pharmacies, higher education institutions, even scientists and healthcare professionals are still waiting for the legalization of cannabis for medical and scientific purposes in Ukraine. SWOT analysis shows that present business structures, educational and scientific institutions, regulatory bodies, and the competency of domestic specialists are strong enough to develop a new market of cannabis-based medicinal products in Ukraine. However, a long-term ban on medical cannabis requires more time for creating the entire ecosystem. This market can be quite attractive in Ukraine. It is characterized by high growth rates, low entry barriers and a substantial demand. Yet, its advancement depends significantly on the appropriate regulatory framework, high level of awareness among health professionals and society as a whole, and involvement in scientific study to become a part of the global medical cannabis market.

**Discussion:**

The holistic approach is aimed to improve health and life quality of Ukrainian patients through cannabis-based medicinal products. It consists of three components: changes in legislation and regulation procedures; changes to value orientations in society; observance of stakeholders’ interests and purposes. Specific recommendations are worked out to realize this approach in Ukraine.

## Introduction

At present, the medical cannabis market is one of the fastest-growing and most perspective markets worldwide. As of 2019, it was estimated at $3.5 billion, and according to the 2025 forecast, a stable growth up to $20.2 billion is expected, with the compound annual growth rate (GAGR) index of 24.4% (Industry Research 2019). By 2020, more than 50 countries have legalized cannabis and cannabinoids for medical purposes, including Canada, most of the US, Germany, Luxemburg, Switzerland, Uruguay, Australia, etc. In Ukraine, the process of national legislation liberalization concerning cannabis application for medical purposes is at a nascent stage. It is, therefore, appropriate to conduct a complex analysis of stakeholders’ targets, expectations and possible actions, evaluate opportunities and risks for this market and develop recommendations for suitable conditions for its sound development.

The **purpose** of the study is to form a holistic approach aimed at establishing a comprehensive environment to develop the market of cannabis for medical purposes and, consequently, improve life and health quality of Ukrainian patients.

### Tasks of the research

- to carry out a general study of legal and regulatory norms regarding medical cannabis in the USSR and former Soviet Republics, including Ukraine;
- to study pharmacy students’ opinion on the feasibility of medical cannabis legalization in Ukraine and their awareness of its therapeutic properties;
- to analyze the aims and interests of the key stakeholders of the perspective medical cannabis market in Ukraine, as well as its strengths and weaknesses, opportunities and threats, by applying strategic analysis tools;
- under a holistic approach, offer recommendations to encourage a full-scale groundwork for medical cannabis in Ukraine, backed by legislative initiatives and changes in social value orientations.

## Background

### The study of medical cannabis status in the USSR and ex-USSR countries

Even though, according to the Convention of 1961 and the Convention of 1971, cannabis and cannabinoids can be used for medical and scientific purposes, their enumeration into the Schedules of the highest prohibition degree makes the regulation of these substances considerably harder (United Nations 1961, 1971). The countries of North and South America, most countries of the European Union (EU), and others found various ways of making cannabis-based medicinal products (CBMP) available for patients. However, medical cannabis in the former Soviet Union countries mostly remains inaccessible for patients due to the lack of legalization of cannabinoids for medical purposes and the high-prejudiced population.

To perform complex analysis of the situation regarding cannabis legalization for medical and scientific purposes, it is worth analyzing the use of this plant, both in the Ukrainian Republic as a part of the **the USSR** and in ex-USSR countries these days. Before 1961, cannabis use for medical and scientific purposes was legal in the USSR. However, the free circulation of cannabis, cocaine, opium, morphine, heroin, and other substances was limited, according to the Decree of the USSR Central Executive Committee (CEC) of 1928 “On measures to regulate the trade in narcotic drugs”. There was also a stated list of enterprises allowed to produce and trade in narcotic substances (Fedorov 2008).

In the USSR State Pharmacopoeia of 1948 (eighth edition), composed between 1938 and 1944, there were monographs devoted to descriptions of “Herba *Cannabis indicae*”, “Extractum *Cannabis indicae* spissum” and “Fructus Cannabis” (Pharmacopoeia Committee 1948). These monographs referred to “Herba *Cannabis indicae*” and “Fructus Cannabis” describing their general appearance, microscopic characteristics, numerical indicators, possible impurities, storage requirements, the maximum single and daily dose. As concerns “Extractum *Cannabis indicae* spissum”, preparation, properties, identity tests, purity tests, the maximum single and daily dose were represented. It is worth noting that “Herba *Cannabis indicae*” and “Extractum *Cannabis indicae* spissum” were included into Schedule B – the list of potential medicines, the prescription, dosage and storage of which had to be done with precaution (Pharmacopoeia Committee 1948).

Despite the possibility of *Cannabis indica* cultivation for medical purposes, since 1934, in accordance with the Decree of the USSR CEC, illegal seeding of this plant was covered by an article of the Criminal Code of Ukrainian SSR (Fedorov 2008). After the USSR ratified international conventions – the Single Convention on Narcotic Drugs (1961), the Convention on Psychotropic Substances (1971) and the United Nations (UN) Convention Against Illicit Traffic in Narcotic Drugs and Psychotropic Substances (1988) (United Nations 1961, 1971, 1988)- the turnover of cannabis plant, including for medical and scientific purposes, was completely prohibited. Illegal activity related to seeding of prohibited plants that contained narcotic substances was regulated by the criminal codes of the USSR republics, including the Criminal Code of Ukraine. As a rule, a person was liable to a prosecution in case of repeated actions related to the cultivation of narcotic drug-containing plants within a current year after the imposition of administrative penalty (Korneev 2010).

After the collapse of the USSR, newly independent states completely borrowed the Soviet system of regulation of narcotic and psychotropic drugs flow, where cannabis and cannabinoids were completely prohibited to be used for recreational, medical and scientific purposes. Relatively recently, some ex-USSR countries began to liberalize legislation to regulate the flow of these substances.

Today, the only country in the post-Soviet territory, where cannabis and cannabinoids for medical purposes are legalized, is **Lithuania**. On October 11, 2018, the Saeima of Lithuania voted for amendments introduction into the Laws on Pharmacy and Narcotic Drugs and Psychotropic Substances which came into effect on May 1, 2019. Thus, according to legislative changes, physicians are allowed to prescribe registered cannabis and cannabinoids-based medical products only for certain indications: multiple sclerosis, HIV/AIDS, severe forms of epilepsy and oncology diseases (Marijuana Business Daily 2019). It is possible to manufacture, import, export, sell (wholesale or retail) medical products containing cannabis substances after receiving the relevant license. At the same time, the cultivation of cannabis plants in Lithuania remains banned (Aviza 2019). In fact, legalization is allowed only for medicinal products based on herbal or synthetic cannabinoids like Sativex, produced by GW Pharmaceuticals, in a similar way to the majority of EU states. The main disadvantage of this approach is the low economic accessability of cannabinoid-based products, because of their high cost and narrow scope of application. At present, despite the official legalization of cannabis and cannabinoids, preparations based on them are practically unavailable for Lithuanian patients.

**Georgia** decriminalized the use of cannabis. However, it was not legalized for medical purposes. Thus, on November 30, 2017, the Constitutional Court of Georgia completely abolished criminal liability for the use of cannabis, considering this norm to be unconstitutional reference the right for freedom in personal development. Before that, the consumption of cannabis was imposed a penalty for the first time and a criminal liability further on. Later, on November 30, 2017, the Constitutional Court of Georgia ruled out an administrative punishment for the use of cannabis to be unconstitutional. Actually, it allowed the individuals aged over 21 to use cannabis, except smoking in public places, which is fined at $300 – $450. Besides, storing of cannabis in Georgia leads to administrative punishment (up to 5 g of dried cannabis) and criminal punishment (if exceeding) (Akhmeteli 2018; Schiller 2018).

In **Estonia**, in spite that cannabis for medical purposes is not legalized, the unregistered medicinal products containing cannabinoids can, nevertheless, be available for patients. Thus, the Estonian State Agency of Medicines (ESAM), on a physician’s advice, can consider a claim on the unregistered import for a definite patient. That is to say, claims on Sativex (Nabiximols), containing herbal cannabinoids, were twice approved by ESAM. Besides, in Estonia, there is no criminal responsibility for cannabis storage up to 7.5 g of dried bud (Marijuana Doctors 2018; European Monitoring Centre for Drugs and Drug Addiction 2018).

In the **Russian Federation,** the Federal Law N 168-FЗ was adopted by the Gosudarstvennaya Duma “On Introducing Changes into the Federal Law “On Narcotic Drugs and Psychotropic Substances”, as Regards the Improvement of Order of Cultivation of Drug-Containing Crops”, which came into effect on July 5, 2019 (The Russian Federation Federal Law 2019). According to this law, it is allowed growing narcotic drug-containing plants for research and scientific purposes, in expert activity for the production of narcotic and psychotropic substances in medicine and veterinary science. This activity can be performed only by the licensed state enterprises obliged to submit annual reports on placement and area of land plots used for cultivation of narcotic drug-containing crops.

However, according to the Decree of the Russian Federation № 101 of 06.02.2020, “On determining strains of narcotic plants permitted for growing to be used in production of narcotics and psychotropic substances for medical and (or) veterinary use, and for industrial growing, not related to production or manufacture of narcotics and psychotropic substances, including requirements to strains and conditions for growing”, only the strains of opium poppy are permitted for medical and veterinary purposes. Cannabis containing maximum 0.1% of tetrahydrocannabinol (THC) is only allowed for industrial growing (The Russian Federation Decree 2020).

It is worth summarizing that patients access to medical cannabis preparations is still limited in ex-USSR countries, despite the legislation liberalization all over the world. Baltic countries (Lithuania, Estonia), being the EU members, take a more active position. The decriminalization of cannabis use in Georgia also testifies to democratic initiatives and actions to observe human rights. At the same time, in other countries of the former Soviet Union, including the Russian Federation, cannabis is under absolute prohibition, both for medical, recreational purposes and for scientific research. Legislation liberalization in these countries is likely to happen only after changes are introduced into the Single Convention on Narcotic Drugs (1961), the Convention on Psychotropic Substances (1971), in line with recommendations suggested at the 41st Expert Committee on Drug Dependence (ECDD) meeting.

### Specific features of the regulation of cannabis and cannabinoids for medical purposes in Ukraine

In **Ukraine**, cannabis and cannabinoids for medical and scientific purposes are prohibited at the legislative level. The regulation of these substances is performed under the Law of Ukraine “On Narcotic Drugs, Psychotropic Substances and Precursors” setting legal and organizational frameworks of the state policy concerning the traffic of above mentioned substances in Ukraine, with cannabis and cannabinoids being part of this group (Law of Ukraine 1995). According to the Decree of the Cabinet of Ministers of Ukraine (CMU) № 770, as of May 6, 2000, “On the Approval of the Schedule of Narcotic Drugs, Psychotropic Substances and Precursors”, cannabis, cannabis resin, cannabis extracts, and tincture are included into Schedule I as “especially dangerous narcotic drugs, with the circulation to be prohibited”, and THC and its isomers – into Schedule II “especially dangerous psychotropic substances, with the circulation to be prohibited” (Decree of CMU 2000).

The application of Cannabis species plant is allowed only for industrial purposes, under Schedule III: “plants containing narcotic and psychotropic substances, the circulation of which is allowed for industrial purposes” (Decree of CMU 2000). Herewith, growing the plant of Cannabis species for industrial purposes is allowed provided that the dried straw of seeds used has the maximum THC content of 0.08%. At this date, certain companies operate in Ukraine dealing with the production of cosmetic products based on technical hemp, food products made of hemp seeds, clothes, equipment for growing technical hemp, plant culturing units, software, etc. (CannaFair 2019). Research activity, new strains cultivation and hemp growing technologies are exercised by the Institute of Bast Crops (2019).

Despite the ban on cannabis and cannabinoids use for medical and scientific purposes in Ukraine, there is a considerable need for preparations based on them. Thus, according to the online Petition “To regulate cannabis for science and medicine means to defend citizens’ constitutional rights”, over 2 million people in Ukraine do not receive any effective pharmaceutical care (Verkhovna Rada of Ukraine 2019). Among them, over 20 thousand children are suffering from drug-resistant epilepsy, hundreds of thousands of cancer patients, over 100 thousand of palliative patients, dozens of thousands of war veterans with post-traumatic stress disorder.

The usage of CBMP will enable satisfying partially the need for pain-relieving preparations, considering a low availability rate of opioid analgesics in Ukraine. According to the analysis of International Narcotics Control Board data, the intake of narcotic opioid analgesics (fentanyl, oxycodone, morphine, hydromorphone, and pethidine) in Ukraine had dropped nearly 30% during 2014–2016, compared to that of 2004–2006: from 93 to 66 daily doses for statistical purposes (s-DDD) per 1.000.000 inhabitants. Ukraine takes the lowest positions among European countries in this matter, taking into consideration that opioid use in many western countries (Austria, Belgium, Denmark, Germany, Netherlands, Spain, Switzerland, USA) exceeds 10.000 s-DDD per 1.000.000 inhabitants (Bosetti et al. 2019; International Narcotics Control Board 2019). According to the data of the Institute of Analysis and Advocacy report, morphine availability rate for patients in various regions of Ukraine constituted 11.2 to 14.3% in 2012–2016 (Institute of Analysis and Advocacy 2018). These indices demonstrate the importance of studying medical cannabis and cannabinoids’ properties, and the need to establish a congenial atmosphere to form the CBMP market in Ukraine.

The issues of medical cannabis legalization are of interest to the Ukrainian public. With this respect, in May 2019, the sociological group ‘Rating’ conducted research in which over 2000 people from all regions of Ukraine took part (Social Group Rating 2019). According to the results, 30% of respondents heartily supported to legalization of medical cannabis, 28% partially supported it. However, a quarter of respondents expressed their strong opposition to this initiative. Most often, respondents approving legalization of medical cannabis and cannabinoids are males aged between 18 and 35, having a high-income level and living in the capital of Ukraine, Kyiv. Opponents of legalization are normally females over 50, with a low-income level, living in the western regions of Ukraine. It stands to reason, that generally a higher education level, broader knowledge, absence of established prejudices influence the positive attitude to medical cannabis legalization.

In recent years, the movement to change the official status of cannabis plant has gained momentum considerably, including the need to liberalize the national legislation. Specifically, Cannabis marches have been held in Ukraine for more than 5 years, bringing up the issues of medical cannabis legalization and its usage decriminalization. The latest march took place in October 2019. It was participated by individual activists, and representatives of over 10 various public organizations representing the interests of oncologic patients, HIV/AIDS patients, people suffering from post-traumatic stress disorder and other diseases. The main demand was to register a new legislative draft providing access to cannabis- and cannabinoid-based preparations for Ukrainian patients (UNIAN Information Agency 2019a). On May 312,019, the II International Medical Cannabis Conference (IMCC 2019) took place covering speakers from the EU countries, the USA, Israel. About 400 specialists, including more than 200 physicians from all over Ukraine, took part in it (Ukrainian Association of Medical Cannabis 2019). However, the online Petition “To regulate cannabis for science and medicine means to defend citizens’ constitutional rights” promoted legislative changes for cannabis and cannabinoids legalization for medical and scientific purposes and garnered the necessary amount of votes.

The main claims of this Petition are as follows: observance of human rights related to use of effective cannabis-based preparations; establishment of adequate conditions for comprehensive application of herbs in medical and scientific activity; the settlement of legal turnover of cannabis-based products (Verhovna Rada of Ukraine 2019). On March 202,019, its public discussion took place in the Committee of the Verkhovna Rada of Ukraine on Issues of Human Rights of National Minorities and Interethnic Relations; however, due to the lack of a quorum they did not take a decision. On May 152,019, this Committee unanimously supported the draft law submitted by nongovernmental organizations “On the introduction of changes to certain legislative acts of Ukraine concerning support of the fundamental human right to life” aimed at cannabis legalization for medical and scientific use (UNIAN Information Agency 2019b). However, the 8th Verkhovna Rada of Ukraine never put this draft law to a vote. As of the date of writing this article, the new bill on legalization of cannabis and cannabinoids for medical and scientific purposes, which is being prepared by the governing coalition in the new 9th Verkhovna Rada, has not been registered yet, and its text is inaccessible for acknowledgement.

## Methods

### Qualitative analysis

Stakeholder and SWOT analysis are most reasonable to reveal the complex effect that legalization of medical cannabis in Ukraine may have for various parties concerned.

Stakeholder analysis is based on the process of systematically gathering and analyzing qualitative information. It will allow identifying and evaluating the expectations, purposes, interests of stakeholders and predicting their possible actions. Kammi Schmeer noted that stakeholder analysis “allows policymakers and managers to interact more effectively with key stakeholders and to increase support for a given policy or program” (Schmeer 2000). Besides, according to Schmeer (2000), if this analysis is conducted before a policy or a program is implemented, policymakers and responsible executives (managers) “can detect and act to prevent potential misunderstandings and/or opposition to the policy or program”. To identify key stakeholders, a stakeholder power-interest grid was used (Eden and Ackerman 1998). According to this grid, stakeholders can be divided into four groups (players, context setter, subjects and crowd) depending on the level of their interests and power. In this paper, the expectations and interests of key stakeholders were analyzed, and their actions after legalization of medical cannabis were predicted.

The next qualitative analysis in this research is the SWOT analysis which can be used as a strategic planning framework while studying both an organization and a particular market or industry. The SWOT analysis is a descriptive tool rather than a prescriptive one, however, it allows shaping a specific vision of the market with due regard for various factors. Thus, the potential market of medical cannabis requires an understanding of the internal strengths and weaknesses, in particular the following ones: existing resources and capabilities and core competencies of the main players. External opportunities and threats enable us to evaluate trends and drivers, the possible competitive situation in the market, as well as the influence of political, economic, technological, legislative, international and other factors affecting the development of the cannabis market in Ukraine (Sammut-Bonnici and Galea 2015).

### Quantitative analysis

#### Sampling justification

To determine the awareness level about the significance of cannabis and cannabinoids use for medical purposes among future specialists in pharmaceutical sphere (general population), the quantitative analysis was conducted, i.e. a survey. As a sampling frame, the School of Pharmacy students at the Bogomolets National Medical University (Kyiv, Ukraine) were selected. To sum up, about 2500 students study at the School of Pharmacy, including full-time and part-time programs. Probability sampling techniques were used, random sampling in particular, which, according to Walliman (2006), allows obtaining reliable data on general population. Besides, random sampling is vital in case when general population has similar characteristics. The sample size in this research comprised 435 persons with the following indices: confidence interval – 97% and confidence level – 5%. In the basic survey, the participants were part-time students – 77.5% and full-time students – 22.5%. Most respondents have incomplete higher education (53.3%) and are fourth-year students. More detailed information on the respondents is given in Table 1.

**Table 1:** Educational characteristics of 435 pharmacy students at the Ukrainian Bogomolets National Medical University who answered a questionnaire about medical cannabis during April to May 2019

Evaluation criteria	Number of respondents	Percentage (%)
*Form of study:*		
Full-time	98	22.5
Part-time	337	75.5
*Year of study*		
1	0	0
2	45	10.4
3	0	0
4	337	77.5
5	52	12
*Education level*		
Higher education	47	10.8
Incomplete higher education	233	53.5
Specialized secondary education in pharmacy	87	20
Specialized secondary education in medicine	67	15.4

It should be observed that the majority of pharmacy students at the Bogomolets National Medical University study part-time to obtain a degree of Specialist (Master) of Pharmacy which directly influences the sample structure. These degrees are practically identical in Ukraine, as a Master’s degree in Pharmacy had been introduced comparatively recently with the purpose of harmonization of national educational standards to international ones. Some students already have higher education (in economics, medicine, biology, philology, etc.), 15.4% of students have already received the specialized secondary education in medicine, and 20% have got the same in pharmacy (a Junior Specialist’s degree). In fact, a Junior Specialist’s degree can be considered as a Bachelor’s degree. In this connection, the majority of students, who already have the specialized secondary education in pharmacy, actually work in pharmacies and receive their higher education part-time (mostly, these are second-year students). Some of the students entered part-time study after graduating from secondary school, and the rest are the full-time students.

Although pharmacy students are not full-rate health care specialists, it is reasonable to study their awareness level and attitude towards the use of cannabis for medical purposes. Since cannabis and cannabinoids are prohibited for medical and scientific purposes in Ukraine, the issues on their therapeutical properties, the endocannabinoid system and others are not considered in curricula and programs to prepare degree-seeking pharmacy students. Therefore, pharmacy practitioners’ awareness respecting this problem is likely to be at the same level as that of students. Besides, the survey included students who mostly study in their final year at the university, they have already had their internship in pharmacies, in accordance with the standards of Specialist (Master) of Pharmacy program. Some part-time students have experience of working in pharmacies. Senior students, who already got the knowledge in physiology, pharmacognosy, pharmacology, etc., consider it important to include materials on cannabis and endocannabinoid system’s properties, and their specific medical use into the educational program. It is doubtless that general population and sampling frame selected for the research cannot provide the appropriate evaluation of awareness and attitude level to legalization of cannabis and cannabinoids for medicine. However, this allow obtaining useful data for holistic approach concerning development of the perspective CBMP market in Ukraine, with the purpose of providing patients with effective pharmaceutical care.

#### Data collection technique

We have developed a questionnaire to study the awareness level of pharmacy students at the Bogomolets National Medical University. The survey took place during April to May 2019. The questions cover such issues as the necessity to provide Ukrainian patients with CBMP, legalization of these products, expediency of conducting this research, incorporation of information on properties of cannabis and cannabinoids in courses for pharmacists, and regulation of these products market. Structure of the questionnaire consists of three parts, namely respondents’ details, main part, and feedback (see the online Appendix). There are two types of questions in the main part. Some questions include a brief analysis of the problems with medical cannabis and cannabinoids to determine the awareness level of respondents or their opinion on different initiatives on CBMP. The answers to these questions are illustrated in the pie and bar charts. Another type of questions rate the importance of certain issues regarding cannabis for medical and scientific purposes on a scale of 1 to 5, where 1 is ‘not important’ and 5 is ‘very important’. The survey results were processed with Microsoft Excel. It is worth saying that the developed questionnaire also bears an educational character. Since the information on CBMP is very contradictory and imperfect in Ukraine, there exist many prejudices as to its use-related risks. Besides, many people, including healthcare professionals, are ill-informed on cannabis and cannabinoids use for medical purposes. Thus, 64.5% of respondents noted that they received useful information on medical cannabis and cannabinoids throughout the survey, and 21.8% noted that they would rather receive this information. Also, 50% of students considered the information obtained to be very important for them, and 35.5% marked it as important.

## Results

### Survey results of students, getting higher pharmaceutical education in Ukraine

It is vital to study this issue among health specialists since Ukraine is now in a process of liberizing its cannabis-related legislation to regulate the use for medicine and science. The Petition “To regulate cannabis for science and medicine means to defend citizens’ constitutional rights” states that there are over 2 million citizens deprived of their access to effective treatment in Ukraine (Verkhovna Rada of Ukraine 2019).

We have examined the respondents’ awareness of the above mentioned matter based on questionnaire taken by 435 Ukrainian pharmacy students at Bogomolets National Medical University during April to May 2019. It is worth noting that other results presented in figures below are also based on the analysis of this survey.

**Fig. 1:**
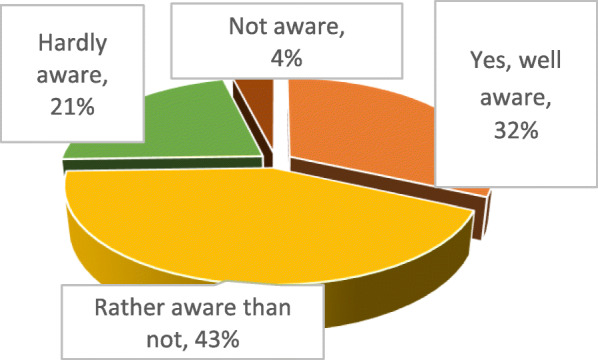
Awareness by Ukrainian pharmacy students of limited access to effective treatment for more than 2 million Ukrainians

Thus, around a third of Ukrainian pharmacy students are well aware that over 2 million Ukrainians have a limited access to effective treatment, 43% confirm they are quite aware, and over 20% of the future pharmacy professionals practically do not know about this problem (Fig. 1). At that, over 75% of respondents agree that it is ‘very important’ to solve this problem.

**Fig. 2:**
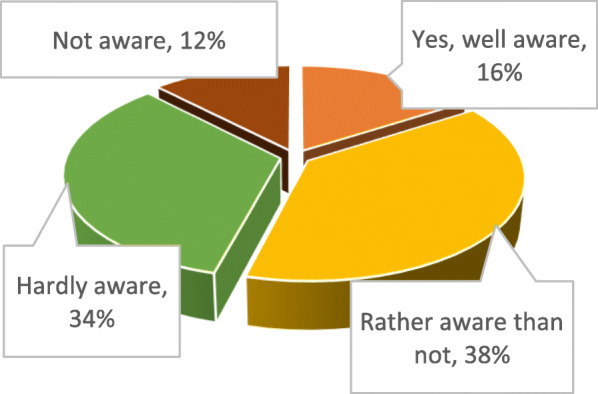
Awareness by Ukrainian pharmacy students of therapeutic properties of cannabis and cannabinoids in treating certain diseases

Only 16% of the polled students are well aware of cannabis and cannabinoids properties for the treatment of certain diseases, while 34% are hardly aware, and 12% are not aware at all (Fig. 2).

**Fig. 3:**
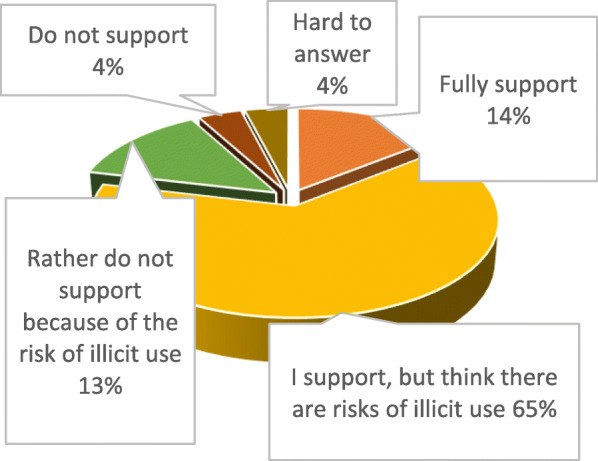
Ukrainian pharmacy students’ attitude to legalization of medical cannabis in Ukraine

More than 65% support legalization of cannabis and cannabinoids for medical and scientific purposes, but, at the same time, they are apprehensive of possible risks, 14% fully support legalization and 13% do not support it (Fig. 3).

Generally, it is possible to conclude that the great majority of future Ukrainian pharmacy specialists have a positive attitude to cannabis plant legalization for medical and scientific purposes. Besides, nearly 44% of respondents have noted that it is ‘very important’ to create a national research basis for studying therapeutic properties of cannabis and cannabinoids.

In many countries where cannabis and cannabinoids are legalized in this or that way, for instance, in Canada, the USA, Israel, academic programs have been elaborated for healthcare professionals regarding properties and medical use of these substances (Pennsylvania Pharmacists Association 2018; Ontario College of Pharmacists 2018). We examined the feasibility of including materials on cannabis, cannabinoids, endocannabinoid system and other aspects in the program for Master of Pharmacy, and obtained positive feedback.

**Fig. 4:**
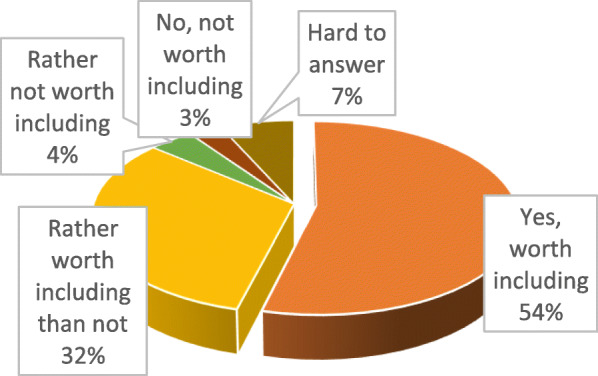
Ukrainian pharmacy students’ attitude to including data on medical cannabis and cannabinoids in academic programs

54% agree that such materials should be included, while 32% consider that they should rather be included (Fig. 4).

Besides, nearly half of the students surveyed (48.5%) think that it is ‘very important’ to compensate for Master of Pharmacy academic programs with studying cannabis and cannabinoids for medical and scientific use.

**Fig. 5:**
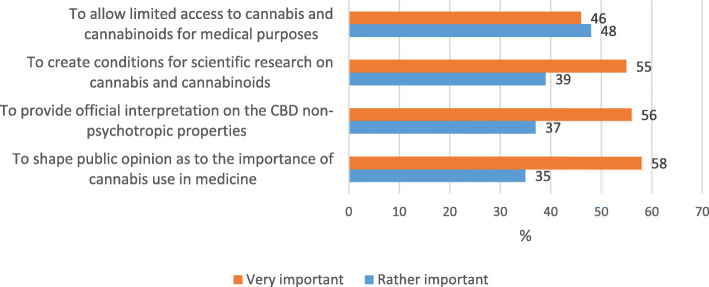
Estimating factors affecting liberalization of Ukrainian legislation concerning cannabis and cannabinoids for medical purposes based on Ukrainian pharmacy students’ opinion

Taking into consideration that the use of cannabis and cannabinoids, including for medicine and science, is completely prohibited, and legislation liberalization is under preparation design, it is vital to evaluate the importance of certain changes in the status of these substances. 58% of the future pharmacy specialists consider a present shaping of public opinion on medical cannabis use to be ‘very important’. 56% consider it is ‘very important’ to advise the key stakeholders on non-psychotropic properties of certain substances in cannabis plant, in particular, those of CBD. This cannabinoid, according to the reports of ECDD, is a harmless and useful substance for human health and public welfare. It has numerous applications in industry, nutrition, cosmetics. It is also used to relieve certain states, for instance, those related to epilepsy (World Health Organisation 2018). 55% of pharmacy students consider it is ‘very important’ to set conditions for a sound scientific research on cannabis and cannabinoids (Fig. 5). On average, about 5% of respondents consider these initiatives to be ‘rather not important’, and a bit over 1% – to be ‘unimportant’.

It should be noted that the above-mentioned points, in the respondents’ opinion, are preliminary stages before cannabis and cannabinoids are legalized for medical purposes, and prospectively – for recreational purposes. They facilitate minimization of possible risks on the part of society and reduce a number of popular prejudices that during a long time cannabis was believed to be a dangerous narcotic drug having no therapeutic effect. In consequence, this will help create the environment for effective and consecutive liberalization of legislation in Ukraine.

**Fig. 6:**
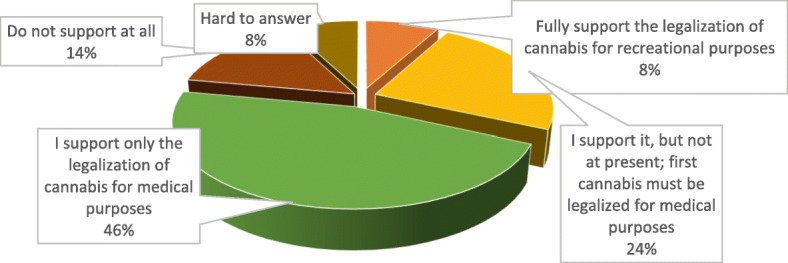
Ukrainian pharmacy students’ attitude to legalization of cannabis and cannabinoids for recreational purposes in Ukraine

Despite that legalization of cannabis for recreational use is not regarded in Ukraine yet, the existing global trends are aimed at this very issue. In this respect, it is vital to learn the future pharmacists’ attitude to cannabis legalization not only for medical but also for recreational purposes. So, according to the research result, the majority of students (46%) support only legalization of cannabis and cannabinoids for medical purposes, and 14% do not support it at all. At the same time, 32% of respondents quite support marijuana legalization, similar to alcohol and tobacco. 8% of them support total legalization and 24% think it would be feasible to legalize marijuana on a phased basis: first for medical and scientific purposes, and only later – for recreational ones (Fig. 6).

It is worth noting that 65% of respondents said they received useful information about medical use of cannabis while filling in the survey form, and 22% confirmed that it was quite useful. At that, 50.5% of future pharmacy specialists agreed that information presented in the survey form was ‘very important’. The results testify that providing information on medical cannabis both for public health professionals and the rest of society is a key and necessary instrument to implement effective liberalization policy in regards to these substances.

### Stakeholder analysis results of the potential CBMP market in Ukraine

**Fig. 7:**
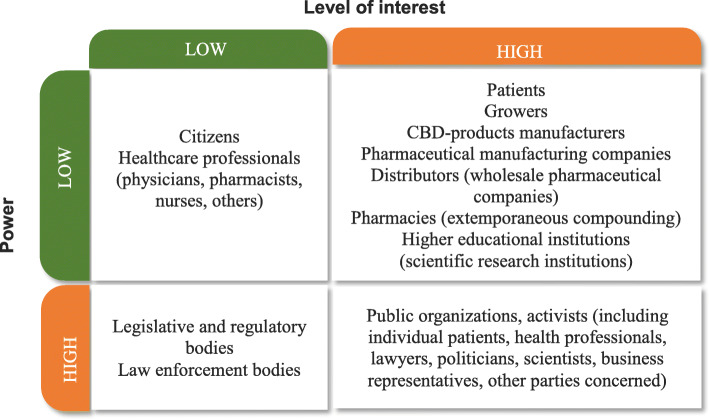
Stakeholder power\interest grid on the potential market of medical cannabis in Ukraine. Note: Healthcare professionals (physicians, pharmacists and others), after medical cannabis legalization, are transferred into the status of stakeholders having high power and low interest, as they have a direct impact on the products accessibility for patients

Legalization of cannabis and cannabinoids for medical and scientific purposes, and consequently, the status change for these substances in Ukraine will involve various stakeholders having different interests on this issue and various level of power and influence. Thus, depending on the power and interest level, we can distinguish several stakeholders in the Ukrainian market of medical cannabis (Fig. 7).

At the stage of providing conditions for legalization of medical cannabis, the key stakeholders are those who possess high power and high interest. Public groups, separate activists and other parties concerned literally ‘push through’ the necessity of legislative regulation of medical cannabis for the benefits of patients. Stakeholders having a high power and low interest level are state policymakers. They do not only possess exclusionary power for legalization but also create conditions to regulate this market.

However, an adequate functioning of the medical cannabis market requires both to identify potential stakeholders and to analyze their expectations and predictable actions after legalization is approved. For more detailed information see Table 2.

**Table 2:** Expectations and predictable actions of the key stakeholders in the medical cannabis market in Ukraine

Expected results	Predictable actions after legalization	Notes
**Stakeholders: high interest / high power**		
*Public organizations (activists): patients, healthcare professionals, lawyers, politicians, scientists, business representatives and other concerned parties*		
Ensuring the interests of patients in obtaining economically and physically available, high-quality, effective, safe medical and pharmaceutical care when using medical cannabis.	Monitoring patients’ rights to receive appropriate medical and pharmaceutical care, cooperation with state regulatory bodies, health professionals, patients and other stakeholders. Creation and support of the stakeholders network to inform and exchange ideas and experience. Carrying out activities for better social awareness of medical cannabis, advantages and risks of its use including activities participated by opinion-leaders (PR-actions, operation of various societies and voluntary organizations).	These stakeholders are a ‘driving force’ at the stage of preparation to the process of medical cannabis legalization, especially concerning stakeholders having a high power but low interest. They possess high power due to their leadership qualities, charisma, the ability of inspiring and uniting people with similar values.
**Stakeholders: low interest / high power**		
*Legislative and regulatory authorities*		
Development and adequate performance of the system that provides affordable and effective medical and pharmaceutical care by using medical cannabis through various tools, such as the Health Technology Assessment (HTA), under the international Conventions’ requirements, the WHO’s recommendations, reports and alerts of the INCB and other international documents.	Creation of legal framework regulating the medical cannabis turnover, also as a component of the general strategy of state narcotic drugs policy, which may include legalization of other drug containing plants and decriminalization of their use. Introduction of amendments and changes to various normative legal documents, for instance, to the Law of Ukraine “On Narcotic Drugs, Psychotropic Substances and Precursors” (Law of Ukraine 1995), as well as to subordinate laws, such as the Decree of CMU №770 (Decree of CMU 2000) and others. Establishing conditions to create new herbal medicinal products or herbal preparations by developing monographs in the State Pharmacopoeia of Ukraine (SPhU). Introducing the mechanisms of cannabis traffic regulation for medical and scientific purposes according to Art. 23 and Art. 28 of the 1961 Convention (United Nations 1961).	The medical cannabis market is new for Ukraine, yet the existing legal and regulatory system can provide regulating instruments, according to the requirements of International Conventions (licensing, quoting, needs assessment, harvest control, etc.). For this reason, it requires some amendments to be introduced on behalf of government, in particular the Ministry of Health of Ukraine (MHU) and the State Service of Ukraine on Medicines and Drugs Control.
*Law enforcement authorities*		
The presence of a legal framework to regulate and prevent the illicit trafficking of cannabis-based preparations, containing narcotic drugs and psychotropic substances.	Providing preventive mechanisms for illicit turnover of medical cannabis containing narcotic drugs and psychotropic substances (herbal substances, herbal preparations or medicinal preparations of herbal origin, both registered and prepared in pharmacy conditions) in accordance with the current legislation of Ukraine.	The lack of decriminalization of cannabis and corruption element in Ukraine may reduce the effectiveness of regulating its illicit trafficking.
**Stakeholders: high interest/ low power**		
*Patients*		
Obtaining physically and economically available, effective and safe medical and pharmaceutical care with the application of CBMP to improve the health and life quality.	Monitoring additional information on cannabis-based preparations for treatment and their official status: cooperation with health specialists (physicians, licensed pharmacists), association into public organizations (groups) for self-assertion as to obtaining accessible medical and pharmaceutical care.	Negative tendencies in Ukraine should be taken into consideration: the average availability level of the ‘golden pain management standard’ – morphine – was between 11.2 and 14.3% during 2012–2016, even despite the liberalization of legislation (Institute of Analysis and Advocacy 2018).
*Growers of cannabis*		
Possibility of official cultivation and growing of cannabis plant, both of outdoor (hemp) and indoor (medical marijuana), with various THC and CBD content, within the legislative environment framework. Legislation liberalization as concerns the growing of hemp. The purpose is to create a sustainable business (investment promotion, gaining a market share, profitability).	Cultivation and growing of cannabis strains with various THC and CBD content for industrial and medical purposes, depending on the requirements of the current legal and regulatory framework. Investment promotion for creating relevant conditions for growing cannabis, especially indoor, personnel preparation and training, safety provision, automation of the process, as well as formation and support of sales markets considering competition, etc.	Additional advantages: new job opportunities, development of business activity, including small businesses, investments promotion, encouraging ‘illicit’ growers to come out of the shadow, etc.
*Manufacturers of CBD-products*		
Liberalization of growing, processing and manufacturing of CBD-products based on the varieties with a low THC-content (their turnover shall not fall into the scope of the law on narcotic drugs and psychotropic substances). Creating conditions for the development of a sustainable profitable business within the legislative environment.	Manufacturing CBD-products in accordance with the legal framework considering the minimum permissible THC level (0.2% in the EU countries, 0.3% in the USA, 1% in Switzerland). Investments promotion: establishing economic and technical conditions for processing cannabis herbal substance, manufacturing and qualified staff training. Creation of identifiable brand, the product portfolio reaching the preferences of target audience, product promotion, formation and realization of a competitive strategy for business development.	The CBD-products market is highly competitive: there are fewer legal limitations, high growth and profit rates and, currently, low entry barriers in the Ukrainian market (an attractive factor for foreign companies). Additional advantages: creating new jobs, business promotion, revenues for local and state budgets, etc.
*Pharmaceutical manufacturing companies*		
The product portfolio diversification due to development and manufacturing of cannabis-based products, including narcotic and psychotropic products, gaining a new market share, profitability improvement, image creation, brand formation. For foreign companies: entering a new market with goods from their product portfolio.	For domestic companies: creation of new medications including narcotic and psychotropic ones (preclinical and clinical trials, formulation of dosage forms and methods of quality control), registration, manufacturing, sales, promotion, pharmacovigilance. For foreign companies: entering a new medicinal products market, their registration (Epidiolex, Sativex) or entering the standardized products market, for instance, of cannabis flowers, including manufacturing of CBMP under pharmacy conditions.	Foreign companies have an advantage compared to domestic ones, which is the time factor and intangible resources including effective promotion of cannabis-based preparations. Their major disadvantage is the product price and low economic accessibility for the Ukrainians.
*Distributors (wholesale pharmaceutical companies)*		
An opportunity of purchasing, storing and selling of CBMP to wholesalers (distributors), retailers (drugstores) and for storing (health care institutions) backed up with licensing. Consequently, there is the entrance into a new market, product range enlargement and profitability.	Purchasing of cannabis-based preparations from manufacturers (foreign and domestic ones), logistics organization, storing, trading (wholesale pharmaceutical companies, pharmacies, healthcare facilities) in accordance with legal requirements, including those for narcotic and psychotropic substances circulation.	These intermediaries are currently interested in the market of registered medicines or of CBMP containing narcotic or psychotropic substances that can be sold only via drugstores.
*Pharmacies (with extemporaneous compounding)*		
Legal opportunity of CBMP manufacturing under the pharmacy conditions in line with current legislation - for instance, cannabis oil from standardized raw materials or dronabinol. Writing monographs on cannabis plant, cannabinoids (THC, CBD), etc. Entering new markets (patients, health care facilities), brand formation, profitability.	Purchasing of cannabis herbal substances for CBMP manufacturing under magistral formulas, establishing economic and technical basis for extemporaneous compounding, drugs form formulations, qualified personnel training, and quality control under the current legislation. Cooperation with physicians, studying of the current legal framework, providing high-quality pharmaceutical care.	Cannabis-based preparations made under magistral formulas have a competitive advantage. They are not subject to state registration in Ukraine. Besides, pharmacies can sell finished pharmaceutical products or dietary supplements containing cannabinoids.
*Higher education institutions (scientific research institutions)*		
Formation of new competencies for students and scientists, adjustment or creation of training programs in accordance with current scientific results (for example, meta-analysis). Obtaining new knowledge through the science and consequently creating scientific products (dissertations, articles, monographs, patents, etc.) to improve lives and society.	Adjustment of training programs and curricula for medical and pharmaceutical higher education institutions (departments) given the scientific-based results on cannabis and cannabinoids therapeutic properties, their application in medical and pharmaceutical practice, etc. Conducting scientific research, creation of new research products, organization of scientific conferences, regular experience exchange. Development of HTA issues and standards (protocols) of treatment.	Higher education institutions are, normally, scientific centers, which allow research results to be integrated into training programs for students. Besides, scientific research institutes can be separately considered as the basis for conducting scientific research.
**Stakeholders: low interest / low power**		
*Healthcare professionals (physicians, pharmacists)*		
For physicians: availability of CBMP with effectiveness evidence base and safety for treating various diseases, existence of treatment protocols and clinical guidelines, legal framework allowing them to prescribe cannabis-based preparations for medical use. For pharmacists (licensed drugstores): existence of legislative framework allowing them to legally purchase, store, produce and sell CBMP, including those with narcotic and psychotropic properties.	Acquiring new knowledge on medical cannabis, modes of action, evidence base, functioning of endocannabinoids system and other information through self-education and training programs. Establishing professional associations, sharing experience and knowledge, studying the legal and regulatory framework on cannabis-based preparations turnover and their availability in various countries.	It is important for state bodies, public organizations and professional associations to take necessary adjustment steps in terms of little interest and low motivation shown by many health specialists. This is determined, among other factors, by insufficient awareness of medical cannabis, prejudices and reluctance to change the legal and regulatory framework.
*Citizens*		
Safety and good order legal protection through the enforcement authorities and prevention of the illicit turnover of CBMP containing narcotic and psychotropic substances.	Compliance with national laws regulating the flow of medical cannabis containing narcotic and psychotropic substances.	These stakeholders are heterogeneous. For instance, a greater focus should be placed on church representatives, separate local communities, as they can have their special purposes, expectations and values.

### SWOT analysis results of the potential CBMP market in Ukraine

Legalization of medical cannabis in Ukraine opens up space for the development of new markets, as it has already taken place in many European countries, the USA, Canada, South American countries and other states. Both CBMP containing a high concentration of TCH and its isomers, and CBD-dominant products, the full-spectrum extracts or broad-spectrum extracts, can be applied for medical use and improve people’s health life quality.

However, it is necessary to evaluate predictable risks and opportunities, as well as strong and weak aspects, what should be the focus of attention of the participants in these markets for effective development of medical cannabis industry in Ukraine. As an effective strategic instrument, the SWOT analysis would suit this best.

So, **Strengths** of the potential market of medical cannabis in Ukraine are as follows:

1. The considerable growth of research articles devoted to use of cannabis and cannabinoids in medical practice: by the end of January 2020, 21.459 papers were published on PubMed, 15.391 of them concerned the influence of cannabis on a human body. During the last 5 years, articles growth on the topic constituted 105%. Besides, the report of the American National Academy of Sciences, the Health Canada review, etc. are accessible, so a considerable experience of medical use of cannabis is obtained (PubMed 2020; Usenko and Kosyachenko 2019).
2. The presence of the potential participants for this market in Ukraine: domestic and foreign pharmaceutical manufacturers, agriculture companies, analytical laboratories, higher educational establishments, scientific research institutions, pharmacies, in particular those having licenses for extemporaneous compounding, wholesale pharmaceutical companies, etc.
3. The current legal and regulatory framework does not require considerable changes. Basically, corrections and amendments are needed to be in line with the international conventions requirements. The positive factor is also liberalization of the national legislation on the circulation of narcotic and psychotropic drugs, aimed at the improvement of their physical and economic availability for patients, in the palliative medicine sphere in particular.
4. The presence of state regulatory bodies which can function as the Cannabis Agency in accordance with the requirements of Art. 23 and Art. 28 of the 1961 Convention (United Nations 1961), and also of the Monitoring center to gain information on narcotic and psychotropic drugs consumption to assess the needs and quota limits.
5. The presence of qualified personnel (specialists of pharmaceutical and agriculture businesses, scientists, teachers, physicians, pharmacists, Health Technology Assessment (HTA) professionals, representatives of state regulatory bodies, etc.) who can practically apply the obtained knowledge and skills in the public health sphere, business, science, education, regulation sphere and so on.

#### Weaknesses

1. The attitude of Ukrainian society towards cannabis is rather constrained. There are prejudices against, for instance, the idea that legalization of medical cannabis can facilitate access to it in the illicit market. According to the survey, 65% of future pharmacy specialists, who support cannabis legalization for medical and scientific purposes, at the same time, believe there is a risk of its illegal circulation.
2. Evidence-based medicine provides insufficient information regarding the efficiency and safety of medical cannabis for treating various diseases. Its data base is quite moderate and weak so far. Clear evidence relates to cannabis and cannabinoids use in the treatment of muscle spasm in patients with multiple sclerosis, neuropathic pain and intractable childhood epilepsy (Barnes and Barnes 2016; European Monitoring Centre for Drugs and Drug Addiction 2018).
3. The absence of clear and unified classification and terminology concerning medical cannabis: there are different definitions to describe the products containing cannabis and cannabinoids and allowed for medical purposes in various countries. Besides, the additional findings are required regarding the scientific research on cannabis plant, interoperation of phyto- and endocannabinoids and other biologically active substances in a human organism (Health Canada 2018).
4. Low availability of complete and actual data on therapeutic efficiency, possible advantages and risks of cannabis and cannabinoids use for medical and scientific purposes. Thuswise, according to the International Narcotics Control Board (INCB) report of 2018, the main factors limiting controlled substances availability for patients are the lack of knowledge, addiction development concern, including that of health professionals (International Narcotics Control Board 2018).
5. Due to a complete statutory ban on cannabis use for medical and scientific purposes in Ukraine, there is almost no practical experience of its cultivation, manufacturing, production, conducting scientific research, etc. As a consequence, the effective functioning of national participants on new potential markets depends also on the time scale. The absence of articles (monographs) in SPhU about cannabis plant and cannabinoids is a restrictive barrier to the production and quality control of CBMP, including an extemporaneous production under pharmacy conditions.
6. There is a big risk of fungus diseases and microbiological contamination for cannabis plant. These factors are of critical importance for providing the needed quality of herbal substance and, consequently, finished herbal product (Greenwell 2012). Besides, growing of cannabis strains aimed at obtaining a herbal product with a high rate of CBD and/or THC suggests using only female plants: cultivation of a crop containing male plants reduces the rate of cannabinoid contents considerably (Spitzer-Rimon et al. 2019).

#### Opportunities

1. About 2 million people in Ukraine, according to the Petition “To regulate cannabis for science and medicine means to defend citizens’ constitutional rights”, suffer from diseases that can be relieved by CBMP. Among them are patients with chronic pain, epilepsy, post-traumatic stress disorder, multiple sclerosis and other illnesses (Verkhovna Rada of Ukraine 2019).
2. The use of cannabis for medical purposes can affect the consumption rate of other preparations, opioid analgesics, in particular, taking into consideration the research results and experience of other countries (Wiese and Wilson-Poe 2018; Khan et al. 2019). However, considering a low availability rate of opioids (morphine, fentanyl) in Ukraine, using medical cannabis will allow patients not to substitute, but rather to compensate for their deficit (Institute of Analysis and Advocacy 2018).
3. CBMP markets are attractive: fast growth rate, high demand index, possibility of differentiating the product portfolio for different market segments, relatively low barriers for entering a new market, which, as a consequence, influences the formation of sustainably profitable business (Grand View Research 2019). Besides, the emerging new markets facilitate formation of new workplaces, development of small and medium businesses and budget revenues.
4. Market legalization, concerning cannabis-based products for medical purposes, will facilitate a reduction of illicit volume. As for patients, quality standardized products on a doctor’s recommendations will be available. Potential market participants, for instance, plant growers, will be able to ‘come out of the shadows’ and run their business activity on a legal basis (Global Commission on Drug Policy 2016).
5. Decriminalization of use and storing cannabis in a limited quantity not for sales can be good for patients because legalization of CBMP, especially in the initial stage, does not always presuppose physical and economic availability of these products. Besides, decriminalization will ease the burden on the state penal setting of Ukraine: 80% of criminal law violations relating to narcotic and psychotropic substances trafficking comprise their use and storage, and not sales. Among narcotic and psychotropic substances seized by law enforcement authorities during 2018, over 70% was cannabis (Center for Mental Health and Drugs and Alcohol Monitoring of Ministry of Health of Ukraine 2019).

#### Threats

1. Legislative and regulatory restrictions related to CBMP in Ukraine can considerably reduce their availability for patients, as well as the investment appeal of potential markets. However, a non-regulated or poorly regulated market, the CBD-products market in particular, can initiate distribution of low quality products, aggressive promotion and consumers cheating on actual cannabinoids content and other negative after-effects (U.S. Food and Drug Administration 2019).
2. Despite the World Health Organisation’s (WHO’s) recommendations to transfer cannabis, cannabis resin, extracts and tincture of cannabis, as well as of THC and its isomers to less regulated Schedule of the 1961 Convention, the UN Commission on Narcotic Drugs has not yet taken a positive decision on this issue (Walsh et al. 2019). This factor plays a negative role in liberalization of medical cannabis in Ukraine on the part of state policymakers.
3. The lack of one-for-all approach to regulate the world cannabis products market, including the medical cannabis market (for instance, the definition of maximum allowable THC rate in various countries is based rather on political decisions than on scientific ones) (Clark et al. 2012). Each country independently chooses approaches to regulate medical cannabis flow: the majority considers Conventions’ requirements, and some states which legalized cannabis for recreational purposes (Canada, Uruguay, some states of the USA) even contradict them.
4. Foreign registered medicinal products containing both herbal and synthetic cannabinoids (dronabinol, nabiximols, nabilon, CBD) are usually characterized by a high cost which can influence their economic availability for Ukrainian patients (Aliekperova 2019). Besides, the CBMP market belongs to emerging markets which is characterized by enhanced activity with intellectual property (Hahn 2019). This allows Big Pharma to strengthen their competitive positions, which will have a negative effect on the development of a domestic market (Prohibition Partners 2019). Thus, starting with the early 1990s, pharmaceutical giants like AbbVie, Sanofi, Merck, GW Pharmaceuticals started the process of patent covering of the results relating to cannabinoids’ effect on a human organism (Reuters Graphics 2019).
5. Considering a low availability of opioid analgesics in Ukraine because healthcare specialists (physicians, pharmacists) are not ready to prescribe and dispense them in a necessary quantity for patients, there are risks relating to medical cannabis too (Institute of Analysis and Advocacy 2018). These risks include health professionals having lack of motivation, necessary knowledge, skills and qualification concerning these preparations. An insufficient evidence base is another risk factor in prescribing preparations of medical cannabis.
6. The existence of illicit cannabis market in Ukraine threatens development of the medical cannabis market, especially at initial stages: established channels and distribution system, more affordable prices, obtaining the product without prescription, absence of bureaucratic procedures, etc. However, without a systematic approach to drug policy it is impossible to give up a ‘total ban’, according to recommendations of the International Commission of Drug Policy (Global Commission on Drug Policy 2018, 2019).

## Discussion

**Fig. 8:**
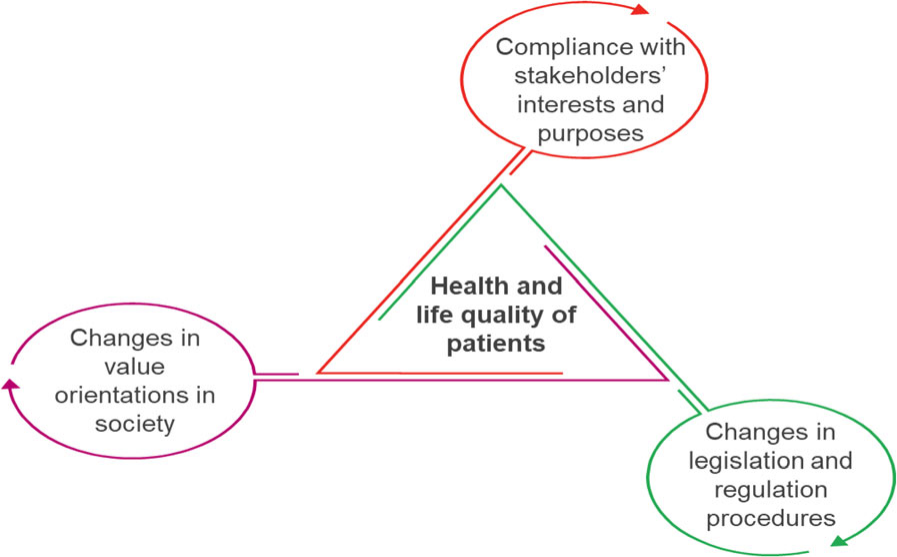
Holistic approach to full supply of medical cannabis to Ukrainian patients

To provide physical and economic availability of CBMP for patients suffering from certain diseases, it is necessary to apply a holistic approach making a system consideration, with regards to possible changes and transformations. This approach is based on three key elements: legislative base and regulatory mechanisms, interests and values of the key stakeholders, and changes to value orientations in society. Together they will allow improving health and life quality indicators of patients (Fig. 8).

Given that we have already analyzed the interests, expectations and anticipated actions of stakeholders in the potential market of medical cannabis in Ukraine, we focus on approaches to form the legal framework and regulation mechanisms for this market and to change value orientations in society. These approaches have a strategic character and are developed based on instrumental analysis results (for instance, the stakeholder analysis and SWOT analysis), as they determine the direction and specifics of this market development.

### Approaches and recommendations reference to legal framework formation

1. To establish a state strategy respecting narcotic and psychotropic substances to develop the one-for-all approaches to regulating the turnover of these substances, including cannabis and cannabinoids in Ukraine. In its formation, it is advisable to target on both the international human rights conventions providing the right to life, the highest attainable standard of physical and mental health, be free from discrimination and mental health and social and economic rights (International Drug Policy Consortium 2016), and the information reported by Global Commission on Drug Policy (GCDP). For instance, the reports mentioned focus on the regulations-change paradigm for narcotic and psychotropic substances: both the ‘total ban’ and the absence of adequate regulation facilitate development of the illicit drug market (Global Commission on Drug Policy 2019).
2. In accordance with Art.16 of the Law of Ukraine “On Narcotic Drugs, Psychotropic Substances and Precursors”, to set the course of development of new narcotic and psychotropic preparations, including those containing cannabis and cannabinoids by means of regulatory by-laws (Law of Ukraine 1995). Attention should be paid to conflicts of laws: for instance, illicit drugs seized by law enforcement authorities cannot be used later for the production of legal medicinal products. According to Ukrainian legislation they must be utilized. It does not just help improve access to narcotic and psychotropic substances for Ukrainian patients and refine income deficiency for pharmaceutical companies, it also leads to additional state expenses on imported raw materials and utilization of the illicit substances.
3. In accordance with the approved state strategy concerning narcotic drugs, to develop a new law on cannabis or introduce relevant amendments to the Law of Ukraine “On Narcotic Drugs, Psychotropic Substances and Precursors”. This is believed to contribute to legal activity related to cannabis and cannabinoids use for medical and scientific purposes (Law of Ukraine 1995). This law must consider the requirements of Art. 23 and Art. 28 of the Single Convention on Narcotic Drugs (1961), in particular, the foundation of a state cannabis agency with specified functions. This includes stating the soil lots for cannabis cultivation for the production of narcotic drugs, granting licenses, crop purchasing and its further distribution, liability for export and import, needs estimation, submission of statistic information to INCB and other functions (United Nations 1961).
4. Ukraine already has efficient mechanisms to implement the requirements of international conventions on drugs control. Thus, the State Service of Ukraine on Medicines and Drugs Control can perform as a cannabis control agency. More particularly, it refers to its structural branch. The Directorate of state regulation and control deals with circulation of narcotic drugs, psychotropic substances and precursors and counters their illicit turnover. This branch is responsible for lisensing and quoting the cultivation of narcotic drug-containing plants, identifying needs in narcotic substances, collection of statistics and its submission to international structures/bodies and others. Besides, the Center for mental health and drugs and alcohol monitoring at the MHU has been functioning in Ukraine since 2009. Its functions cover both the monitoring of drugs and collecting information on use of narcotic products (Institute of Analysis and Advocacy 2018).
5. To introduce changes into the Decree of CMU №770 in May 6, 2000 “On the Approval of the Schedule of Narcotic Drugs, Psychotropic Substances and Precursors” (Decree of CMU 2000) in accordance with recommendations approved at the ECDD WHO 41st session (WHO 2018). And specifically, to exclude cannabis, cannabis resin, extracts and tincture of cannabis from Table I, Schedule 1 “Especially dangerous narcotic drugs, with the circulation to be prohibited” and to include cannabis and cannabis resin into Table II, Schedule 1 “Narcotic drugs, with the circulation to be limited”, and to include extracts and tinctures of cannabis into Table III, Schedule 1 “Narcotic drugs, with the circulation to be limited; in relation to these substances the exceptions to some control measures shall be allowed”. Besides, to exclude THC (isomers and their stereochemical variants) from Table I, Schedule 2 “Especially dangerous psychotropic substances, with the circulation to be prohibited”, as well as dronabinol and its stereoisomers (Δ9-THC) from Table II, Schedule 2 “Psychotropic substances, with the circulation to be limited” and to include them into Table II, Schedule 1.
6. To exclude from the Schedules of narcotic drugs, psychotropic substances and precursors of the Decree of CMU № 770, May 6, 2000, cannabis with the total THC content not exceeding 1.0% and CBD with admixed THC not exceeding 1.0%, because there is no psychotropic effect and the risk of drug addiction development (the case of Switzerland and Uruguay). CBD preparations with THC content of 1.0% have a far stronger therapeutic effect for medical use than a ‘pure’ CBD (entourage effect) (Russo 2018). To obtain official interpretation or advisement from the state bodies of Ukraine (MHU) regarding that preparations containing pure CBD are not subject to control under Conventions regulating the traffic of narcotic drugs.
7. To approve necessary subordinate acts allowing policy implementation for medical cannabis turnover: Decrees of CMU, Orders of MHU, etc. To develop relevant monographs on cannabis plant and cannabinoids in SPhU, treatment protocols, clinical guidelines and other regulating documents. With the help of HTA tools to substantiate the importance of medical cannabis application for treating certain symptoms or diseases, in particular through budget funds or health insurance.
8. To provide a comprehensive framework for conducting research on cannabis and cannabinoids by scientific research and higher education institutions of all types of ownership without licenses and quotas (for a definite number of plants). Considering requirements of the national legislation on medicinal products to allow research and higher educational institutions to cultivate cannabis, to develop medicinal substances and products based on cannabis and cannabinoids, to carry out preclinical and clinical trials.
9. To introduce changes to the Criminal Code and the Code of Administrative Offences of Ukraine to decriminalize the possession and storing of cannabis in a limited quantity without intention to sell it, taking into consideration information provided in Art. 3, p. 2 of the UN Convention on countering illicit turnover of narcotic drugs and psychotropic substances (1988) (United Nations 1988), materials laid in the GCDP reports (Global Commission on Drug Policy 2018, 2019).

### Approaches to the formation of value orientations in society

1. To shape public opinion on the significance of cannabis and cannabinoids use for medical and scientific purposes both among specialists (doctors, pharmacists, scientists), and Ukrainian citizens: 58% of pharmacy students from the Bogomolets National Medical University has noted that it is ‘very important’, and 38% – that it is ‘rather important’. Moreover, it is worth focusing on the principal difference between recreational and medical cannabis. Because of prejudices, lack of knowledge and negative convictions in the society there may occur marginalization of potential patients (Bottorff et al. 2013).
2. A significant role in shaping public opinion on medical cannabis is played by active participation of various stakeholders, in particular by public organizations, opinion leaders among representatives of science, health care, business, politics, other spheres of public activity. For this, various instruments of communication with the public should be used, considering the reach of audience, impact effectiveness and the possibilities of integration to increase a desirable result. For instance, for the second year in a row, the International Cannabis Conference is held in Ukraine. There are physicians, scientists and patients from the USA, Czechia, Switzerland, Germany, Portugal, France, Spain, Israel among the speakers experienced in practical application of cannabis-based products and with information on the achievements of contemporary science (Ukrainian Association of Medical Cannabis 2019).
3. To develop and put into practice informational training programs in educational institutions (schools, colleges, universities) concerning importance of using narcotics-containing plants as medicinal products for the treatment of certain symptoms and diseases, with a focus on the risks of their application for recreational purposes, especially by children and teenagers.
4. Among the professional community, in particular among the representatives of the health care system, who to a large extent determine availability of medicinal products and CBMP for patients, it is necessary to run awareness campaigns concerning cannabis plant, cannabinoids, their properties and advantages, usage risks, interaction with endocannabinoid system, etc. Such campaigns can be organized in the form of practical seminars, lectures, courses, congresses, conferences, working sessions including those with the participation of state bodies. It will result in the development and publishing of recommendations and guidelines, conference, articles, monographs and other scientific and practical materials.
5. Cannabis plant should be considered in complex speaking of high attractiveness of potential cannabis markets. It can be applied in various economic industries, different from health care, beauty and well-being, like building materials (hempcrete), seed-pressed oils for paints and sealants, biocomposites, green energy and others.(Riboulet-Zemouli et al. 2019). To implement the win-win strategy, value orientations should be formed in a society that can create long-term benefits for all stakeholders.
6. Creation of new cannabis-related spheres and markets will directly influence various social and economic indices in the country, especially if high value-added products are manufactured. It will improve humans and pets’ health and life quality, create additional workplaces, promote the entrepreneurship, science, innovative technologies, and consequently provide a steady revenue for local and state budgets.

## Limitations

The survey of cannabis and cannabinoids legalization for medical purposes in Ukraine has covered only the pharmacy students from the Bogomolets National Medical University, which certainly does not present a full picture of the health specialists’ opinion on the issue studied. However, considering its small size, the research is representative and reflects the attitude towards the medical cannabis problem among the students seeking a Specialist’s or Master’s degree in Pharmacy. In reviewing the literature, we have analyzed only those countries’ legislation with medical cannabis and cannabinoids legalized that are the main players in the market of these products. Besides, this analysis is quite workaround and superficial, as it lies beyond the aims of this research. It is necessary for preparing recommendations to develop the relevant legal framework in Ukraine which would enable a decent development of the medical cannabis market. This article also presents the stakeholder and SWOT analyses results. However, since the market of these products is still prohibited in Ukraine, it is rather difficult to identify and evaluate all concerned parties, their goals, interests, expectations, as well as weak and strong points of a potentially new branch and opportunities and threats of its development.

## Conclusions

To improve the quality of life and health of patients suffering from various diseases, it is vital to consider the issue of legalization of cannabis and cannabinoids for medical and scientific purposes, as it has already been done in many countries worldwide. However, to provide adequate conditions for developing these products market and consequently its physical and economic availability for patients, it is advisable to apply a complex approach to study this situation. In this view, we have studied the present accessibility of medical cannabis for patients in the USSR and former USSR countries such as Lithuania, Georgia, Estonia, Russian Federation, and Ukraine. We conducted a survey among pharmacy students to evaluate the anticipation of the future healthcare professionals in Ukraine about cannabis legalization for medical purposes and its adequate use to improve the quality of life and health of patients. So, according to the survey results, the vast majority of respondents demonstrate a positive attitude towards legalization of medical cannabis. At the same time, slightly less than half of the students are practically not informed about the therapeutic properties of these products. Nearly 90% of students think that educational materials on cannabis and cannabinoids use for medical purposes should be included in training programs. They should also include risks and advantages of such application, functioning of endocannabinoid system etc. Using a holistic approach, we performed the stakeholder and SWOT analysis to provide recommendations for an adequate supply of medical cannabis for the patients in Ukraine. It reveals the existing legal framework and regulation mechanisms, compliance with stakeholders’ interests and expectations, as well as formation of value orientations in society speaking.

## Supplementary information

**Additional file 1.** Survey Questionnaire.

## Data Availability

The datasets used and/or analyzed during the current study are available from the corresponding author on reasonable request.

## Abbreviations

CBD: Cannabidiol
CBMP: Cannabis-based medicinal products
CEC: Central Executive Committee
CMU: Cabinet of Ministers of Ukraine
ECDD: Expert Committee on Drug Dependence
ESAM: Estonian State Agency of Medicines
EU: European Union
GAGR: Compound annual growth rate
GCDP: Global Commission on Drug Policy
INCB: International Narcotics Control Board
HTA: Health Technology Assessment
MHU: Ministry of Health of Ukraine
SPhU: State Pharmacopoeia of Ukraine
SWOT analysis: Strength, weakness, opportunity, and threat analysis
THC: Tetrahydrocannabinol
UN: United Nations
USSR: Union of Soviet Socialist Republics
WHO: World Health Organization
