# Supplementary material for: Perspectives on formation of medical cannabis market in Ukraine based on holistic approach
Source: J Cannabis Res. 2020 Oct 2;2:33. doi: 10.1186/s42238-020-00044-y (PMC7819340; doi:10.1186/s42238-020-00044-y)
Supplement: Supplementary file 1 — Additional file 1. Survey Questionnaire. [file 42238_2020_44_MOESM1_ESM.doc]

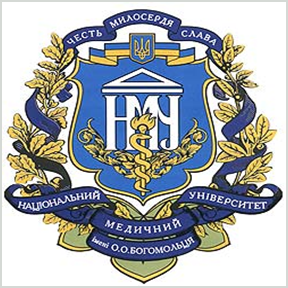
**BOGOMOLETS NATIONAL MEDICAL UNIVERSITY**

**Dear students, we will be thankful for your answers regarding the level of your awareness on the issue of medical cannabis and the relevance of its legalization in Ukraine**

| **Part 1 Respondent’s details** |
| --- |

**Form of education**

Full-time Part-time

**Year of study**

1 2 3 4 5

**Education level**

Incomplete higher education

Specialized secondary education in medicine

Specialized secondary education in pharmacy

Higher education

| **Part 2 Main part** |
| --- |

| **Today, about two million Ukrainians are deprived of the necessary healthcare, including children with pharmresistent forms of epilepsy, oncology patients, palliative patients, war veterans with post-traumatic stress disorder, etc.** |
| --- |

Please, state your awareness of this problem:

Yes, I am well aware of this problem

I am rather aware than not

I am hardly aware

I am not aware

| **Please rate the importance of available and effective medical (pharmaceutical) care for the stated groups of Ukrainian patients on a scale of 1 to 5 (1– not important; 5 – very important)** |
| --- |

**1 2 3 4 5**

| **Medicines made of cannabis compounds are legalized and actively used in the USA, Canada, the EU countries to relieve patients from chronic pains, multiple sclerosis, severe forms of epilepsy, depression, sleep disorder. Continuously, research is conducted as to creating evidence base for the use of these compounds for medical purposes** |
| --- |

Examples of medicines based on cannabis and cannabinoids

| 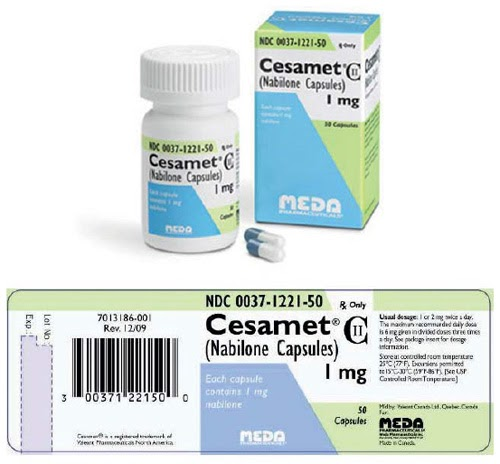**Cesamet** (Nabolon) capsules 1,0 mg | **Marinol** (Dronabinol) capsules 2.5 mg, 5 mg and 10 mg  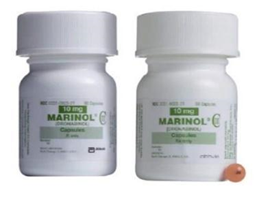 |
| --- | --- |

| **Sativex** (Nabiximols) 1 dose contains 2.7 mg THC and 2.5 CBD  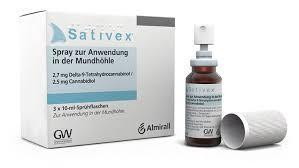 | **Epidiolex** (Cannabidiol) oral solution containing 100 mg/ml  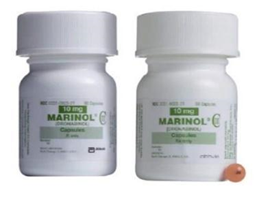 |
| --- | --- |

Please state your awareness of cannabis-based medicinal products properties in the aforesaid conditions:

Yes, I am well aware of the properties

I am rather aware than not

I am hardly aware

I am not aware

| **Please rate the importance of scientific research on creation and enhancing of evidence base of medical use of cannabis compounds on a scale of 1 to 5 (1– not important; 5 – very important)** |
| --- |

**1 2 3 4 5**

| **In Ukraine cannabis, cannabis resin, cannabis extracts and tincture are included in Schedule 1 “especially dangerous narcotic drugs, with the circulation to be prohibited” under the DCM №770. Scientific research of these compounds is also prohibited, although some cannabis compounds, e.g. cannabidiol (СBD), have no psychotropic properties at all and are effective for treating severe forms of epilepsy** |
| --- |

Please state your attitude to legalization of cannabis compounds for medical purposes in Ukraine:

I fully support

I support, but think there are risks of illicit use

I rather do not support because of the risk of illicit use

I do not support

Hard to answer

| **Please rate the importance of creation of a national scientific base as to the medical use of cannabis compounds and the production of cannabis-based medication on the basis of domestic pharmaceutical companies on a scale of 1 to 5 (1– not important; 5 – very important)** |
| --- |

**1 2 3 4 5**

| **In many countries where cannabis and cannabinoids are legalized, for instance, in Canada, the USA, Israel, academic programs have been elaborated for public health specialists regarding properties and medical use of these substances. In Ukraine, educational programs studying the use of cannabis and cannabinoids for medical purposes do not exist at the moment, because of legislative prohibition** |
| --- |

In your opinion, should the materials as to the properties and clinical use of cannabis compounds be included in academic programs for obtaining a Bachelor’s or Master’s degree in Pharmacy in Ukraine?

Yes, worth including

Rather worth including than not

Rather not worth including

No, not worth including

Hard to answer

| **Please rate the importance of creating academic programs for studying cannabis compounds for medical purposes and of preparation of relevant specialists (after legalization) on a scale of 1 to 5 (1– not important; 5 – very important)** |
| --- |

**1 2 3 4 5**

| **On March 20, 2019, public discussion of the online Petition “To regulate cannabis for science and medicine means to defend citizens’ constitutional rights” took place in the Committee of the Verkhovna Rada. The main claims of this Petition are as follows: observance of human rights related to use of effective cannabis-based preparations; establishment of adequate conditions for comprehensive application of herbs in medical and scientific activity; the settlement of legal turnover of cannabis-based products** |
| --- |

Please rate the importance of these changes for regulating patient access to cannabis-based medicinal products in Ukraine:

|  | Very important | Rather important | Rather not important | Not important |
| --- | --- | --- | --- | --- |
| To allow limited access to cannabis and cannabinoids for medical use |  | | | |
| To create conditions for scientific research on cannabis and cannabinoids |  | | | |
| To provide official interpretation on the CBD non-psychotropic properties |  | | | |
| To share public opinion as to the importance of cannabis use in medicine |  | | | |

| **In Canada, Uruguay, some US states (Colorado, Washington, etc.) marijuana is allowed not only for medical, but also for recreational purposes** |
| --- |

Please state your attitude to legalization of cannabis compounds not only for medical, but also for recreational purposes:

I fully support the legalization cannabis for recreational purposes

I support it, but not at present; first cannabis must be legalized for medical purposes

I support only legalization of cannabis for medical purposes

I do not support at all

Hard to answer

| **Part 3 Feedback** |
| --- |

| **Please state if you have obtained useful information as to the problem of legalization of cannabis compounds for medical purposes in Ukraine, while filling in the questionnaire** |
| --- |

Yes, I did

I rather did

I rather did not

No, I did not

Hard to answer

| **Please rate the use of the information you obtained after working with the questionnaire on a scale of 1 to 5 (1– not important; 5 – very important)** |
| --- |

**1 2 3 4 5**
